# Genomic diversity and biosynthetic capabilities of sponge-associated chlamydiae

**DOI:** 10.1038/s41396-022-01305-9

**Published:** 2022-08-30

**Authors:** Jennah E. Dharamshi, Natalia Gaarslev, Karin Steffen, Tom Martin, Detmer Sipkema, Thijs J. G. Ettema

**Affiliations:** 1 Department of Cell and Molecular Biology, Science for Life Laboratory, Uppsala University, SE-75123 Uppsala, Sweden; 2 Department of Pharmaceutical Biosciences, Biomedical Center, Uppsala University, SE-75123 Uppsala, Sweden; 3 Laboratory of Microbiology, Wageningen University and Research, 6708 WE Wageningen, The Netherlands

**Keywords:** Symbiosis, Phylogenetics, Metagenomics, Comparative genomics, Microbiome

## Abstract

Sponge microbiomes contribute to host health, nutrition, and defense through the production of secondary metabolites. *Chlamydiae*, a phylum of obligate intracellular bacteria ranging from animal pathogens to endosymbionts of microbial eukaryotes, are frequently found associated with sponges. However, sponge-associated chlamydial diversity has not yet been investigated at the genomic level and host interactions thus far remain unexplored. Here, we sequenced the microbiomes of three sponge species and found high, though variable, *Chlamydiae* relative abundances of up to 18.7% of bacteria. Using genome-resolved metagenomics 18 high-quality sponge-associated chlamydial genomes were reconstructed, covering four chlamydial families. Among these, *Candidatus* Sororchlamydiaceae shares a common ancestor with *Chlamydiaceae* animal pathogens, suggesting long-term co-evolution with animals. Based on gene content, sponge-associated chlamydiae resemble members from the same family more than sponge-associated chlamydiae of other families, and have greater metabolic versatility than known chlamydial animal pathogens. Sponge-associated chlamydiae are also enriched in genes for degrading diverse compounds found in sponges. Unexpectedly, we identified widespread genetic potential for secondary metabolite biosynthesis across *Chlamydiae*, which may represent an unexplored source of novel natural products. This finding suggests that *Chlamydiae* members may partake in defensive symbioses and that secondary metabolites play a wider role in mediating intracellular interactions. Furthermore, sponge-associated chlamydiae relatives were found in other marine invertebrates, pointing towards wider impacts of the *Chlamydiae* phylum on marine ecosystems.

## Introduction

Porifera (i.e., sponges) are ubiquitous filter-feeding metazoans that provide essential ecosystem services. These animals have complex, deeply integrated, and essential microbiomes that play important roles in processes such as nutrient cycling [1–4]. The sponge microbiome also produces secondary (or specialized) metabolites that may contribute to host chemical defence [5]. Generally, sponges are known as a source of novel secondary metabolites with medical and industrial relevance [6–9]. With increasing exposure to anthropogenic threats, further investigation of the sponge microbiome is essential for understanding host impacts, from acting as detrimental opportunists to providing resilience against dysbiosis and disease [4, 10]. Despite cultivation efforts, many sponge-associated microbial groups remain uncultured to date [11, 12], and have only recently been explored through cultivation-independent sequencing approaches [13–17]. In a recent large-scale survey, sponge-associated microbial communities were shown to be diverse, yet structured, and composed of taxonomic groups with both generalist and specialist host ranges [18]. One of these generalist phyla is *Chlamydiae*, which is found at high relative abundance in some sponge species [19, 20]. Yet, the genomic diversity of sponge-associated chlamydiae has not been previously investigated.

*Chlamydiae* is a bacterial phylum of obligate eukaryotic endosymbionts well-known for animal pathogens, such as *Chlamydia trachomatis* and other *Chlamydiaceae* [21–24]. Many *Chlamydiae* members instead infect microbial eukaryotes and have more extensive metabolic repertoires [21–23]. Chlamydial environmental distribution and abundance has been underestimated, as their small-subunit (SSU) rRNA genes are often missed by primers used to survey microbial diversity [25–27]. However, with the recent use of metagenomic and single-cell approaches sequenced chlamydial genomic diversity is quickly expanding, resulting in a widening view of the potential lifestyles of uncultivated chlamydial groups [27–32]. Retrieving additional genomes affiliated with the *Chlamydiae* is needed to further our understanding of chlamydial ecological impacts, range of host interactions along the parasite-mutualist continuum, and the evolution of endosymbiosis and pathogenicity in this major bacterial group within the *Planctomycetes, Verrucomicrobia*, *Chlamydiae* (PVC) superphylum [33, 34].

The sponge species *Halichondria panicea*, *Haliclona oculata*, and *Haliclona xena*, sampled from an estuary in the Netherlands, were previously found to have high relative abundances of *Chlamydiae* [20]. In the present study, we performed genome-centered analyses of the microbial communities from these sponge species in order to gain insight into chlamydial sponge-associated lifestyles. Comparative analyses of 18 resulting high-quality sponge-associated chlamydiae draft genomes revealed degradative genes found in other sponge symbionts. In addition, these *Chlamydiae* genomes share metabolic features with other members from their respective chlamydial families, and encode genes absent in known chlamydial families composed strictly of animal endosymbionts (i.e., *Chlamydiaceae* and *Parilichlamydiaceae*). Unexpectedly, we also identified extensive genetic potential for secondary metabolite biosynthesis across the *Chlamydiae* phylum. Finally, we found that relatives of these sponge-associated chlamydiae are also associated with additional sponge species and other marine invertebrates. Together, our results suggest that *Chlamydiae* members play ecological roles that impact animals found in marine ecosystems and represent an untapped source for secondary metabolite discovery.

## Results and discussion

### Specific *Chlamydiae* lineages vary in relative abundance across three sponge species

**Fig. 1:**
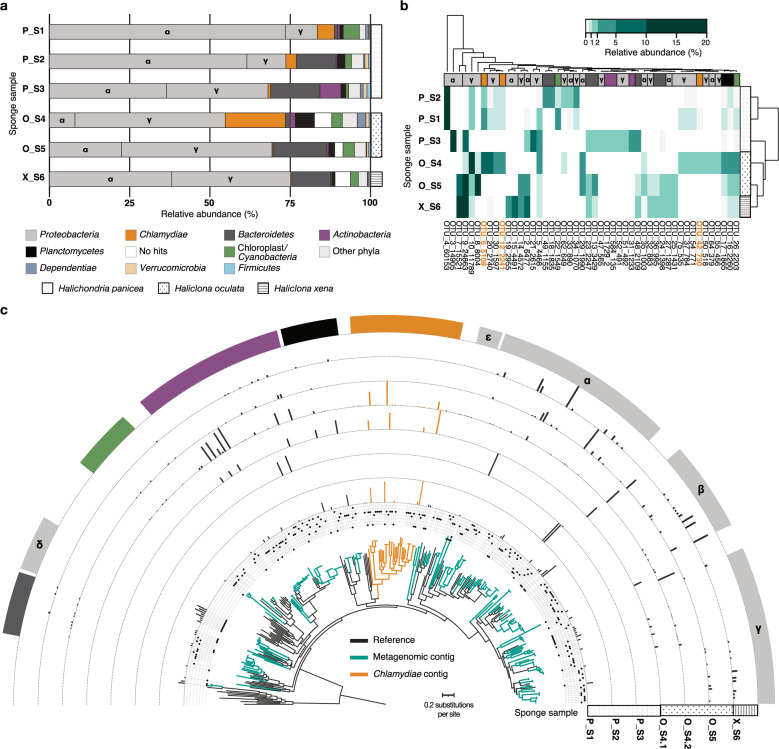
Bacterial SSU rRNA gene amplicon sequencing and metagenomes reveal variably high relative abundances of *Chlamydiae* across sponge specimens. **a** Relative abundances of phyla from SSU rRNA gene amplicon sequence data with ≥1% relative abundance in a sample. **b** Heatmap of OTUs with ≥1% relative abundance in a sponge sample from SSU rRNA gene amplicons. Both OTUs and sponges are hierarchically clustered based on presence patterns. **c** Concatenated maximum likelihood phylogenetic tree of contigs encoding ribosomal proteins (≥5) from across sponge metagenomic assemblies in the context of bacterial reference taxa. The tree is rooted by an archaeal outgroup. The metagenomic origin of each sequence is indicated by the dot plot, with lines corresponding to samples in the following order: P_S1, P_S2, P_S3, O_S4.1, O_S4.2, O_S5, and X_S6. Bars indicate the relative coverage of each ribosomal protein-encoding contig in each metagenome. Phylum affiliation is indicated for each clade according to the colour legend in panel (**a**). In addition, classes are indicated for *Proteobacteria*: *Alphaproteobacteria* (α), *Betaproteobacteria* (β), *Deltaproteobacteria* (δ), *Epsilonproteobacteria* (ε), and *Gammaproteobacteria* (γ). See legend in panel (**a**) for phylum colour assignment and patterns corresponding to each sponge species. See Data S1 for sample information, Data S2 for amplicon OTUs, Data S3 for relative abundances and read counts, Data S4 for metagenomic ribosomal protein contigs and their corresponding coverage, and Data S5 for the ribosomal protein phylogenetic tree with sequence accessions.

Using bacteria-specific SSU rRNA gene amplicon sequencing, high relative abundances of *Chlamydiae* were found in the sponges *H. panicea* P_S1 (5.3%), *H. panicea* P_S2 (3.3%), and *H. oculata* O_S4 (18.7%) (Fig. 1a), which had been collected during the same sampling event as a prior study [20] (Fig. S1 and Data S1). However, *Chlamydiae* relative abundance was substantially lower in three additional sponge specimens, *H. panicea* P_S3 (0.80%), *H. oculata* O_S5 (0.31%), and *H. xena* X_S6 (0.34%) (Fig. 1a), that were collected from a similar location, but at different dates (Fig. S1 and Data S1). This variation was unexpected, as consistently high relative abundances of *Chlamydiae* were previously found across all three sponge species [20]. Nevertheless, *Chlamydiae* were not detected in other studies of the *H. panicea* microbiome [35]. Beyond *Chlamydiae*, bacterial compositions across the sponge species were consistent with a prior investigation, with the same phyla represented and *Proteobacteria* as the dominant phylum (Fig. 1a) [20]. SSU rRNA gene amplicon sequences were subsequently clustered into operational taxonomic units (OTUs) at species-level (97% identity) (Data S2). This revealed variability in the specific bacterial OTUs present in different sponge specimens (Fig. 1b). However, three specific *Chlamydiae*-affiliated OTUs with high relative abundances (up to 8%; Data S3) were found in all samples with higher overall abundances of *Chlamydiae* (P_S1, P_S2, and O_S4) (Fig. 1a, b). Yet, these OTUs were largely undetected in amplicon data from the other sponge specimens (P_S3, O_S5, and X_S6) (Data S3). In a prior study, these same chlamydial lineages were found at high relative abundance across all specimens of the three sponge species, but were largely undetected in surrounding seawater [20]. Together, these results support a close association of these specific chlamydial lineages with two of the investigated sponge species (*H. oculata* and *H. panicea*), while prior work suggests associations with all three sponge species [20].

*Chlamydiae* have also been detected with varying presence and abundance across a diverse range of other sponge species [18]. Despite this, sponge-associated chlamydiae have not been previously investigated at the genome level. With the aim of exploring sponge-associated chlamydial genomic diversity, we sequenced and assembled seven high-quality metagenomes from the six sampled sponge specimens, with two generated for O_S4 (O_S4.1 and O_S4.2) (Fig. S1 and Data S1). We assessed microbial diversity in the resulting metagenomes by identifying contigs encoding ribosomal protein gene clusters, and thus representing distinct microbial lineages. Ribosomal protein sequences from each contig were concatenated and a maximum-likelihood (ML) phylogenetic tree reconstructed (Fig. 1c and Data S4–S5). The relative abundance of each microbial lineage was inferred by comparing coverage of these contigs from each sponge metagenome (Fig. 1c and Data S4). These analyses confirmed the larger patterns in sponge microbial community composition seen in the SSU rRNA gene amplicon results. However, several phyla had lower (i.e., *Bacteroides*, *Cyanobacteria*, and *Proteobacteria*) and higher (i.e., *Actinobacteria* and *Chlamydiae*) relative abundances in metagenomes than amplicons, perhaps due to differences in SSU rRNA gene copy number [36]. Overall, the metagenomes confirmed the chlamydial patterns seen in amplicon data, with three distinct *Chlamydiae*-affiliated lineages found with high relative abundance when *Chlamydiae* were present (P_S1, O_S4.1, and O_S4.2) (Fig. 1c and Data S4–S5).

Together, investigations of sponge-associated microbial communities from amplicon and metagenome data support variable associations of diverse chlamydial lineages with several sponge species. These observed differences in *Chlamydiae* relative abundance indicate that chlamydial presence is non-essential across these sponge species and were unexpected, given that healthy sponges typically have stable microbiomes [4]. Several different factors could be affecting *Chlamydiae* abundance patterns and we are unable to resolve between them here. Variation between samples from the same species could stem from persistent, and thereby still stable, associations of these chlamydial lineages with specific sponge individuals or populations. Conversely, it could suggest temporal changes in the presence of these chlamydial lineages, as a result of seasonality or specific environmental conditions. It is also possible that sponge-associated chlamydiae remain present, but only increase in abundance under specific environmental conditions. As part of their conserved lifestyles, *Chlamydiae* members have both an intracellular dividing phase and an extracellular non-dividing phase where they remain as elementary bodies [21, 24]. *Chlamydiae* members can also enter states of persistence inside host cells when under environmental stress and refrain from dividing [24]. Thus, sponge-associated chlamydiae may still be present in all sponge individuals, but remain at low abundances as elementary bodies or in persistence states. In addition, variation in presence and abundance could also be the result of these chlamydiae infecting a different eukaryotic host that is itself associated with the sponges.

### Genome-resolved metagenomics of sponges expands sequenced chlamydial diversity

**Fig. 2:**
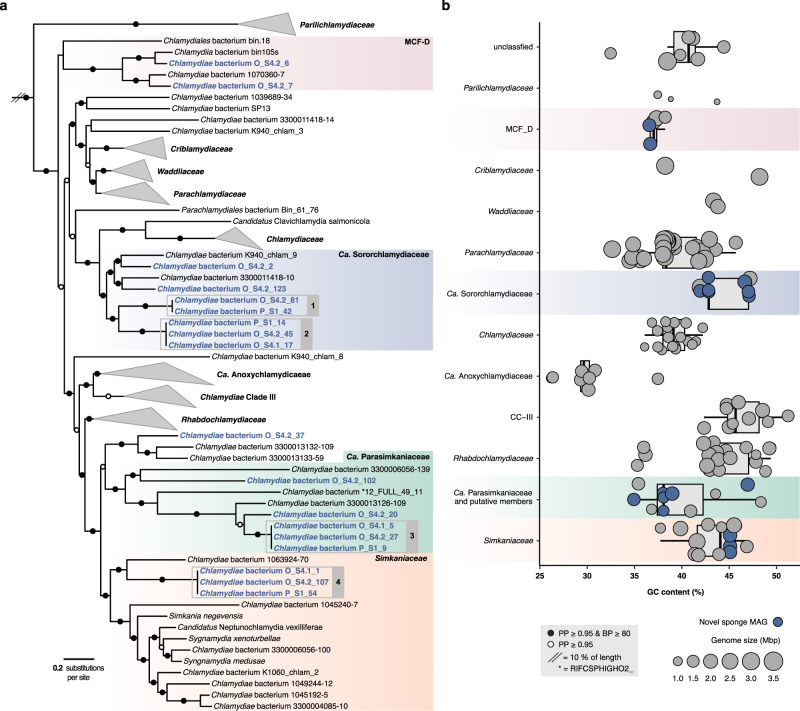
*Chlamydiae*-affiliated metagenome-assembled genomes (MAGs) retrieved from sponges are phylogenetically diverse. **a** Concatenated Bayesian phylogeny of *Chlamydiae* species relationships inferred using 15 marker gene NOGs under the CAT + GTR + Γ4 model of evolution. Branch support is indicated by coloured circles and includes posterior probability (PP) from the Bayesian inference and non-parametric bootstrap support (BP) from a maximum-likelihood reconstruction of the same dataset inferred with the PMSF approximation of the LG + C60 + F + R4 model of evolution (Data S5). Family names are indicated and those not including sponge-associated chlamydiae MAGs collapsed. Sponge-associated chlamydiae MAGs retrieved in this study are highlighted in blue, while chlamydial species groups with ≥95% average nucleotide identity (ANI) are indicated by grey boxes numbered 1–4. The tree is rooted by a PVC outgroup (not shown). See Fig. S3 and Data S5 for additional species trees and uncollapsed phylogenies. **b** Genome characteristics of sponge MAGs (blue) in the context of species representatives from across *Chlamydiae* (grey). Boxplots indicate the distribution of GC content across different chlamydial families, with the area of circles indicating genome size. See Data S6 for genome characteristics and ANI.

To gain further insight into the *Chlamydiae*-affiliated diversity associated with these sponge species, we then used genome-resolved metagenomics to retrieve microbial genomes. Metagenome-assembled genomes (MAGs) were obtained from each of the seven sponge metagenome assemblies using differential coverage profiles and consensus results from several binning tools (Fig. S1). This resulted in 106 medium to high-quality MAGs (median 89% completeness and 1.4% redundancy) (Data S4). MAGs affiliated with *Chlamydiae* were further collected, manually curated, and reassembled (from P_S1, O_S4.1, and O_S4.2; Fig. S1). This resulted in 18 high-quality draft *Chlamydiae* genomes with high contiguity (median 35 contigs), high completeness (median 98.7%), and low redundancy (median 1.01%) (Data S6). Exceptionally, *Chlamydiae* bacterium O_S4.1_1 and O_S1_54 were 99% complete and retrieved on only three contigs each. Phylogenomic trees were then inferred to determine the placement of sponge-associated chlamydiae MAGs within the *Chlamydiae* phylum, using four subsets of concatenated marker proteins with chlamydial and outgroup species representatives (Figs. S2–S3, and Data S6–S7). The obtained species tree topology was consistent across ML reconstructions and the placement of sponge-associated chlamydiae MAGs was highly supported (Figs. 2a and S3, and Data S5). A Bayesian phylogenetic tree was also inferred (Fig. 2a) using the smallest subset of marker proteins (*n* = 15), which included those that best resolved the phyla present in the dataset (Fig. S2 and Data S7). Here, species topology was overall consistent with the ML trees, with a few exceptions for long-branching taxa.

**Fig. 3:**
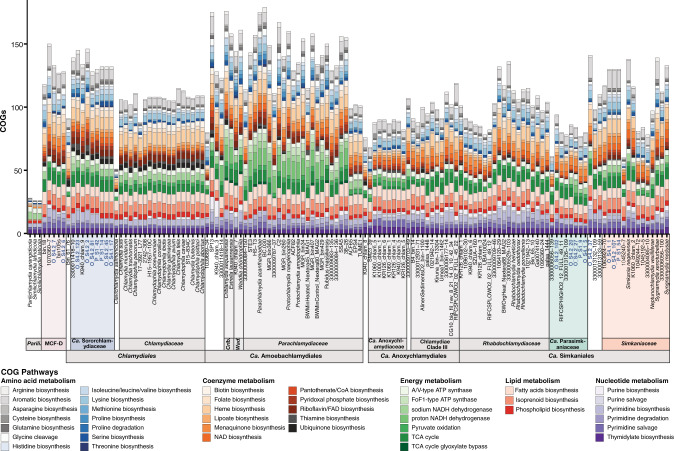
Clusters of orthologous group (COG) pathways across *Chlamydiae* indicate that sponge-associated chlamydiae have similar metabolic profiles to other members of their respective families. Bars are coloured according to COG category and show the number of COGs identified from the pathway in the given genome. COG categories with similar profiles across *Chlamydiae* genomes were excluded but can be found in Fig. S5 and Data S9. Sponge-associated chlamydiae MAGs are in blue, with relevant families coloured accordingly. Order and family names are indicated at the bottom with the following short forms: *Criblamydiaceae* (Crib.), *Parilichlamydiaceae* (Parili.), and *Waddliaceae* (Wad.). See Fig. S5 for an overview of relative pathway completeness and Data S9 for an overview of each COG across *Chlamydiae* genomes.

Sponge-associated chlamydiae MAGs were placed in four distinct chlamydial families (Fig. 2a and Fig. S3). Two MAGs were affiliated with the recently described Metagenomic Chlamydial Family D (MCF-D) [31], which includes a MAG from the glass sponge *Vazella pourtalesii* [37], indicating a more widespread association of this family with sponges (Fig. 2a). Three additional MAGs and *Chlamydiae* bacterium 1063924-70 [31] formed a well-supported clade with *Simkaniaceae* from marine, coastal, and host-associated environments, including invertebrates (Fig. 2a and Fig. S3, and Data S6) [31, 38, 39]. However, five other MAGs consistently form a group sister to *Simkaniaceae* with several uncharacterized freshwater chlamydiae (Fig. 2a and Fig. S3, and Data S6) [31, 40]. Based on its well-supported phylogenetic position and conserved gene content, we propose this *Simkaniaceae*-like sister clade as a new chlamydial family *Candidatus* Parasimkaniaceae (Figs. 2, 3 and Figs. S4–S5). A few long-branching taxa, including one sponge-associated MAG (*Chlamydiae* bacterium O_S4.2_37) were part of the *Ca*. Parasimkaniaceae, with high support in the Bayesian tree, yet clustered together with *Simkaniaceae* in ML phylogenies (Fig. 2a and Fig. S3). Additional genomes are needed to confidently resolve whether these putative *Ca*. Parasimkaniaceae members are indeed affiliated with this family or instead with *Simkaniaceae*.

The remaining seven sponge-associated chlamydiae MAGs formed a clade with other chlamydial MAGs with unknown hosts that were obtained from marine sediment [27] and fungal mycelium [31] metagenomes. This group shares a common ancestor with *Chlamydiaceae* and was previously referred to as *Chlamydiae* Clade IV (Fig. 2a and Fig. S3) [27]. We propose to name this family *Candidatus* Sororchlamydiaceae on the basis of its consistent phylogenetic position, improved taxon sampling in our study, distinct genomic characteristics such as genome size, and conserved gene content (Figs. 2b, 3 and Figs. S4–S5). Identifying sponge-associated *Ca*. Sororchlamydiaceae also has potential evolutionary implications. *Ca*. Sororchlamydiaceae share a common ancestor with exclusively animal-associated chlamydiae including *Clavichlamydia salmonicola*, a fish pathogen [41], and the *Chlamydiaceae* family, that have thus far only been obtained from tetrapods (e.g., mammals, birds, and reptiles) [42]. If many *Ca*. Sororchlamydiaceae are indeed sponge symbionts, this could indicate an ancestral association of these chlamydiae from the *Chlamydiales* order with *Metazoa*, and subsequent long-term evolution with animal hosts.

Overall, the sponge-associated chlamydiae MAGs affiliated with four different chlamydial families (Fig. 2a). Among these, were four phylogenetically distinct chlamydial species groups with a high level of relatedness in species phylogenies and with at least 95% average nucleotide identity (ANI) (Fig. 2a and Data S6). Two of these species’ groups affiliated with *Ca*. Sororchlamydiaceae, and one each with the *Simkaniaceae* and *Ca*. Parasimkaniaceae (Fig. 2a). A representative from each group was obtained from both the *H. panicea* (P_S1) and *H. oculata* (O_S4.1 and O_S4.2) metagenomes, which had high overall *Chlamydiae* relative abundances (Fig. 1a). In addition, SSU rRNA genes from two of these species’ groups corresponded to abundant OTUs identified in the amplicon data (*Ca*. Sororchlamydiaceae species group 2 to OTU_6_5108 and *Simkaniaceae* species group 4 to OTU_42_730; Fig. 2a and Data S4). No SSU rRNA genes were obtained in *Ca*. Parasimkaniaceae species group 3 MAGs. However, we suspect that they correspond to OTU_2241 based on the affiliation of this amplicon sequence with other *Ca*. Parasimkaniaceae members in an SSU rRNA gene tree (Data S5). Together, our results present a widened view on sequenced chlamydial genomic diversity with the addition of 18 high-quality genomes affiliated with the *Chlamydiae* phylum. Furthermore, this expanded diversity has allowed us to resolve and describe two additional chlamydial families, the *Ca*. Sororchlamydiaceae and *Ca*. Parasimkaniaceae.

### Sponge-associated chlamydiae gene content corresponds to family affiliation

To gain information about the putative lifestyles of these sponge-associated chlamydiae and potential for sponge interactions, we then performed comparative genomics analyses to investigate their gene content. We found that within chlamydial families a large proportion of gene content is shared and that few genes are unique to sponge-associated lineages, with more genes shared between closely related species (Fig. S4). Thus, the sponge-associated chlamydiae accessory genome corresponds more to phylogenetic affiliation than to an ecological association with sponges. This also indicates that sponge-associated chlamydiae have similar lifestyles to other chlamydiae. Supporting this, sponge-associated chlamydiae genomes encode hallmark genes associated with endosymbiosis and the typical chlamydial lifecycle that are found conserved across *Chlamydiae* [27, 31]. These include nucleotide transporters (NTTs) that can be used for energy parasitism of ATP, the UhpC transporter that can be used to uptake host-derived glucose-6-phosphate, the transcription factor EUO that acts as the master regulator of the chlamydial biphasic lifecycle, and a type III secretion system that can be used to mediate host interactions through the secretion of effectors [21, 23] (Data S8). The presence of these genes strongly suggests that sponge-associated chlamydiae are likewise endosymbionts with the potential for an intracellular lifestyle in sponge hosts. Chlamydiae could be actively acquired from the surrounding water by the filtering action of the sponge. *Ca*. Sororchlamydiaceae genomes also encode flagellar genes (Data S8), in contrast to most other chlamydiae [27, 32], which they could use to encounter new sponge hosts.

We then compared the presence of central metabolic pathways across *Chlamydiae*-affiliated genomes to see if sponge-associated chlamydiae MAGs resembled previously sequenced groups in terms of core metabolism. Indeed, metabolic profiles of sponge-associated chlamydiae were comparable to other members from the same chlamydial families (Fig. 3 and Fig. S4–S5, and Data S9). More generally, central metabolic pathway presence and completeness was similar across chlamydial families, with some exceptions. Our results mirrored prior findings [21, 29], with protist-infecting chlamydiae (i.e., *Ca*. Amoebachlamydiales) genomes encoding more complete metabolic pathways, and *Chlamydiaceae* and *Parilichlamydiaceae* genomes encoding fewer, consistent with their specialized lifestyles as pathogens with specific animal hosts (Fig. 3 and Fig. S5, and Data S9). Relative to other chlamydial families, *Ca*. Sororchlamydiaceae and MCF-D members encode a larger diversity of genes for the *de novo* biosynthesis of coenzymes and unlike most chlamydiae they encode asparagine biosynthesis genes. Several *Ca*. Sororchlamydiaceae genomes also encode more genes for pyrimidine biosynthesis than most other chlamydiae (Fig. 3 and Fig. S5, and Data S9).

Despite generally conserved core metabolism, genes from certain metabolic pathways are more common in sponge-associated chlamydiae genomes, such as genes involved in *de novo* biosynthesis of aromatic amino acids (*e.g*., tryptophan) in sponge-associated *Simkaniaceae* (Fig. 3 and Fig. S5, and Data S9). It has been suggested that members of the sponge microbiome exchange aromatic amino acids [43] and sponge-associated *Simkaniaceae* may likewise do so or provide them to the sponge host. Similarly, most sponge-associated *Ca*. Parasimkaniaceae encode a sodium-transporting NADH dehydrogenase, which is absent in all other members of the family (Fig. 3 and Fig. S5, and Data S9). Conversely, the first three genes of the TCA cycle (i.e., citrate synthase, aconitase, and isocitrate dehydrogenase) are absent in most sponge-associated *Ca*. Parasimkaniaceae genomes and present in other family members (Data S9). These genes are also absent in *Chlamydiaceae*, which depend on the uptake of host-derived TCA cycle intermediates [21] and this could likewise be the case for sponge-associated *Ca*. Parasimkaniaceae. Overall, sponge-associated chlamydiae metabolic profiles were not convergent, and instead, these chlamydiae largely resembled relatives from affiliated families. Similarly, sponge-associated chlamydiae genomes did not resemble known chlamydial groups thus far composed exclusively of animal pathogens (i.e., *Chlamydiaceae* and *Parilichlamydiaceae*).

### Degradative genes indicate potential for interactions between *Chlamydiae* members and sponges

**Fig. 4:**
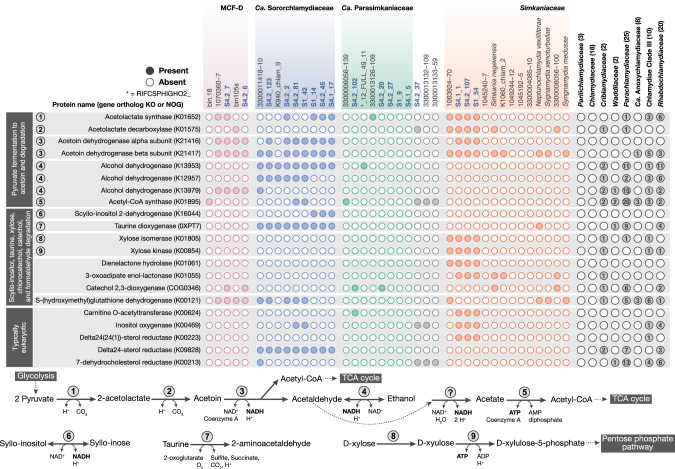
Sponge-associated chlamydiae genomes encode genes related to fermentation, degradation, and typical of eukaryotes. The presence of selected enriched genes across *Chlamydiae* genomes from families with sponge-associated members is shown, alongside a schematic overview of pyruvate fermentation to acetoin, and genes related to degrading acetoin, scyllo-inositol, taurine, and D-xylose. Protein function and gene orthologs are indicated to the left alongside numbers corresponding to the schematic overview. The number of representative genomes from other chlamydial families encoding the given gene is indicated to the right out of the total number of genomes considered in parentheses. See Data S8 for a full overview of gene presence across *Chlamydiae* representatives and corresponding gene annotations for sponge-associated chlamydiae.

Genes more common among sponge-associated chlamydiae, relative to other *Chlamydiae* members, were further investigated to provide additional information about their potential lifestyles. This revealed that sponge-associated chlamydiae encode genes related to taurine, inositol, and xylose degradation (Fig. 4 and Data S8), which have previously been found in the genomes of sponge symbionts. Taurine dioxygenase (TauD), an enzyme that degrades the sulfonate taurine to sulfite, is widespread among members of the sponge microbiome who can use it to degrade host-derived taurine [15], which is found in nearly all marine metazoans [44]. *Ca*. Sororchlamydiaceae genomes encode *tauD* (Fig. 4 and Data S8), suggesting they can use host-derived taurine to acquire sulfite. However, *tauACB* ABC transporter genes (K15551, K15552, and K10831) were not identified (Data S8). This mirrors recent findings of more widespread identification of *tauD* among sponge-associated MAGs than *tauACB* [15]. Nevertheless, in *Ca*. Sororchlamydiaceae members we did identify components of the larger gene families that include sulfonate ABC transporters (COG1116 and COG0600), alongside inconsistent identification of functional orthologs (K02051 and K02049) (Data S8). Further investigation is needed to determine whether these putative sulfonate ABC transporters can transport taurine or if chlamydial TauD is instead used to degrade an alternative sulfonate [45]. Inositols are common in eukaryotes and rare in bacteria, and the sponge host has been suggested as the source of inositols for inositol-degrading bacterial symbionts [14, 46, 47]. *Ca*. Sororchlamydiaceae species group 2 MAGs (Fig. 2a) have the genetic potential to degrade scyllo-inositols using a putative scyllo-inositol 2-dehydrogenase (IolW) (Fig. 4 and Data S8). The gene *iolW* has previously been found in sponge symbionts such as *Ca*. Poribacteria members, which reside in the sponge extracellular matrix [14, 47, 48]. *Ca*. Poribacteria members can also degrade the sugar xylose, using the concerted action of xylose isomerase (XylA) and xylulokinase (XylB) [47]. *Simkaniaceae* species group 4 MAGs (Fig. 2a) encode both *xylA* and *xylB* and thus have the genetic potential to degrade xylose (Fig. 4 and Data S8). Genes for degrading taurine, scyllo-inositol, and xylose are rare or completely absent outside of sponge-associated chlamydiae genomes (Fig. 4 and Data S8). Finding these genes in sponge-associated chlamydiae is thus consistent with a sponge-symbiont lifestyle and the use of host-derived compounds.

Eukaryotic-like proteins and domains are thought to mediate host-microbe interactions and corresponding genes are abundant in the genomes of sponge symbionts [49]. Similarly, such genes are abundant across *Chlamydiae*-affiliated genomes [50]. Here, we identified several additional typically eukaryotic genes as enriched in sponge-associated chlamydiae genomes, including sterol reductases and carnitine O-acetyltransferase (Fig. 4 and Data S8). Sterol reductase genes were identified across *Ca*. Sororchlamydiaceae genomes and in *Simkaniaceae* species group 4 MAGs (Fig. 2a). Sterol reductases perform the final step in ergosterol or cholesterol biosynthesis and have previously been found in intracellular bacteria, including several *Chlamydiae* members [51]. In phylogenetic trees of these sterol reductases (K00223/K00213 and K09828), chlamydial sequences branch together with both bacterial and eukaryotic homologs, and could have been acquired by horizontal gene transfer (HGT) from either (Data S5). Carnitine is abundant in animal tissues and in bacteria it is used as an osmoprotectant or metabolized, although bacteria cannot synthesize carnitine *de novo* [52]. Genes related to carnitine degradation have been found in members of the sponge microbiome and carnitine is also abundant in the sponge extracellular matrix [13]. In animals, carnitine is used in the carnitine shuttle where it is reversibly acylated by carnitine O-acetyltransferase to allow fatty acid transport across the mitochondrial matrix for oxidation [52]. Unexpectedly, we also identified carnitine O-acetyltransferase genes (K00624) in *Simkaniaceae* species group 4 MAGs and *Ca*. Parasimkaniaceae member *Chlamydiae* bacterium S4.2_102 (Figs. 2a and 4, and Data S8). In a phylogenetic tree, these chlamydial sequences branch within eukaryotic homologs suggesting that the genes were obtained by HGT from a eukaryotic host (Data S5). We likewise identified the ABC transporter OpuC (K05845 and PF04069) in these same chlamydial lineages, which can uptake osmoprotectants including carnitine [53] (Data S8). However, we did not detect genes for carnitine degradation (Data S8). Together, this suggests that some sponge-associated chlamydiae may obtain host carnitine to use as an osmoprotectant, or potentially, use carnitine O-acetyltransferase to mediate host metabolism or to acquire host fatty acids. Overall, the presence of these eukaryotic-like genes in sponge-associated chlamydiae genomes indicate host interactions and represent potential targets for future studies to elucidate chlamydiae-sponge interactions.

Genes for degrading acetoin were also identified as enriched in sponge-associated chlamydiae genomes and are rare in other *Chlamydiae* members (Fig. 4 and Data S8). Acetoin is a volatile organic compound that some bacteria can use as an energy and carbon storage compound, and which can act as a sole carbon source under glucose-limitation [54, 55]. Chlamydiae affiliated with the *Ca*. Sororchlamydiaceae, MCF-D, and *Simkaniaceae* species group 4 MAGs (Fig. 2a) may be acetoin-degrading bacteria as most encode an acetoin dehydrogenase complex (AcoA-B) (Fig. 4 and Data S8). AcoA-B is used to degrade acetoin to acetaldehyde and acetyl-CoA, with the concerted reduction of NAD^+^ to NADH [54–56]. The resulting acetaldehyde can undergo further fermentation to ethanol by alcohol dehydrogenases, such as those encoded in *Ca*. Sororchlamydiaceae and MCF-D genomes (Fig. 4 and Data S8), acetyl-CoA can enter the TCA cycle, and NADH can be used as a reducing agent in other reactions or enter the electron transport chain (Fig. 4). Some sponge-associated *Ca*. Sororchlamydiaceae and *Simkaniaceae* may also produce acetoin through the fermentation of pyruvate to acetolactate using acetolactate synthase (Fig. 4). Acetolactate is then converted to acetoin spontaneously under aerobic conditions or through the action of acetolactate decarboxylase [54], which is encoded in some *Simkaniaceae* genomes, including sponge-associated members (Fig. 4). Together, these genes suggest that sponge-associated chlamydiae can degrade acetoin as a carbon and energy source, which could be obtained from members of the sponge microbiome.

In addition, several genes involved in degrading organic pollutants and toxic compounds were also enriched in sponge-associated chlamydiae genomes and rare in other *Chlamydiae* members. A putative S-(hydroxymethyl)glutathione dehydrogenase gene was found in several chlamydiae affiliated with *Ca*. Sororchlamydiaceae, MCF-D, and a few other chlamydial families (Fig. 4 and Data S8). This enzyme is involved in oxidizing formaldehyde, a toxic compound that can be produced as an intermediate during methylotrophy [57], genes for which have been found expressed by sponge symbionts [58]. We also identified genes involved in chlorocatechol and catechol degradation (i.e., dienelactone hydrolase, 3-oxoadipate enol-lactonase, and catechol 2,3-dioxygenase) in a few sponge-associated chlamydiae genomes (Fig. 4 and Data S8). Anthropogenic contamination is a growing concern in marine ecosystems, and microbially mediated degradation of such organic pollutants and other aromatics have a branching point at chlorocatechol and catechol [59]. Genes involved in these degradative pathways have previously been found in members of the sponge microbiome, and may also be used to detoxify compounds produced by other microbiome members [60]. Dienelactone hydrolases perform a key step by degrading dienelactones to maleylacetate, which can then be further catabolised before entering the TCA cycle [61, 62]. *Simkaniaceae* species group 4 MAGs (Fig. 2a) and *Chlamydiae* bacterium 1063924-70, which was obtained from a marine environment (Data S6), encode dienelactone hydrolase (Fig. 4 and Data S8). In addition, genes with the dienelactone hydrolase family protein domain (PF01738) are found in most sponge-associated *Ca*. Sororchlamydiaceae genomes (Data S8). Sponge-associated chlamydiae may use these genes to protect themselves, or the sponge host, from toxic compounds, whether they originate from external sources or other microbial community members.

### Widespread potential for secondary metabolite biosynthesis across the phylum *Chlamydiae*

**Fig. 5:**
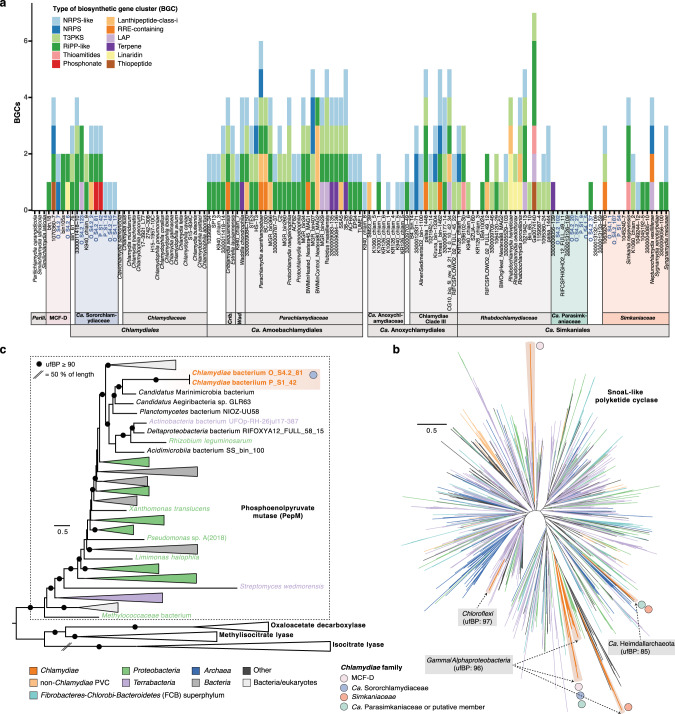
Biosynthetic gene clusters (BGCs) are found across *Chlamydiae* and sponge-associated chlamydiae genomes. **a** The number and type of BGCs (found in >1 genome) identified across *Chlamydiae*. Order and family classification is indicated alongside shortened species names. See Data S10 for a full overview of BGCs. Maximum-likelihood phylogenies of a group of polyketide cyclases (SnoaL-like; PF07366) (**b**), which are involved in secondary metabolite biosynthesis, and of phosphoenolpyruvate mutase (PepM; PF13714) (**c**), which performs the first step in phosphonate biosynthesis. Ultrafast bootstrap support (ufBP) is indicated by a black circle (**c**). Branch and clade colours indicate taxonomy according to the legend (**b**, **c**). The presence of *Chlamydiae* families including sponge-associated chlamydiae genomes is indicated by the coloured circles, with the taxonomy of the sister clade indicated where supported (**b**). See Data S5 for uncollapsed phylogenies and sequence accessions.

Sponges are well-known as reservoirs for the discovery of natural products with medical and industrial importance, many of which are secondary metabolites produced by microbiome members [6–8]. Typically, genes for producing secondary metabolites are organized in biosynthetic gene clusters (BGCs) [7]. Recently, metagenomic analyses have revealed that BGCs are found across a broad phylogenetic range of bacteria in sponge microbiomes [63]. To gain further perspective on the genetic capacity for secondary metabolite biosynthesis across *Chlamydiae*, we used antiSMASH [64] to investigate BGCs in representative genomes and our sponge-associated chlamydiae MAGs (Fig. 5 and Data S10). Unexpectedly, our analysis revealed that BGCs are widespread across the phylum *Chlamydiae*, with many chlamydial genomes encoding multiple and different types of BGCs, most with unknown functions (Fig. 5a and Data S10).

Genomes of *Ca*. Sororchlamydiaceae and *Ca*. Amoebachlamydiales encode the most conserved set of BGCs, with many encoding NRPS-like (non-ribosomal peptide synthetase), RiPP-like (ribosomally synthesized and post-translationally modified peptide), and T3PKS (type III polyketide synthase) BGCs (Fig. 5a). However, BGCs were few in *Ca*. Anoxychlamydiaceae, largely absent in *Ca*. Parasimkaniaceae and sponge-associated *Simkaniaceae*, and completely absent in all *Chlamydiaceae* and *Parilichlamydiaceae* genomes (Fig. 5a). We found additional PKS and NRPS genes, which are central in the biosynthesis of various secondary metabolites, in some sponge-associated chlamydiae MAGs. For example, MCF-D member *Chlamydiae* bacterium S4.2_7 encodes NRPS gene homologs related to those found in *Simkania negevensis* (Data S8). One group of PKS genes, SnoaL-like polyketide cyclases (PF07366), were found encoded in many sponge-associated chlamydiae MAGs (Data S8). Phylogenetic analysis of this protein family showed that it has been gained multiple times by different chlamydial groups and from diverse potential HGT partners (Fig. 5b). Despite BGCs not being widely identified in *Ca*. Parasimkaniaceae, some genomes do encode this PKS gene (Data S8 and Fig. 5b).

Phosphoenolpyruvate mutase (PepM) performs the first committed step for synthesizing fosfomycin and other phosphonates [65]. Fosfomycin inhibits bacterial cell wall biosynthesis by binding to the active site of MurA, which performs the initial step in peptidoglycan biosynthesis [66]. Two *Ca*. Sororchlamydiaceae sponge MAGs (*Chlamydiae* bacterium S1_42 and S4.2_81) encode a phosphonate BGC (Fig. 5a), with closest homology to BGCs used to produce the antibiotic fosfomycin (0.61 similarity score and 50% PepM sequence identity; Data S10). In a phylogenetic tree, chlamydial PepM homologs formed a well-supported clade together with known PepM sequences, indicating that they likely have the same function (Fig. 5c). However, we were unable to determine the HGT donor, since chlamydial homologs formed a clade with diverse bacteria primarily represented by MAGs (Fig. 5c). *Chlamydia* spp. are resistant to very large quantities of fosfomycin due to conserved changes in the MurA active site [67]. Further investigation into MurA sequence evolution and the genetic potential for fosfomycin production across *Chlamydiae* could elucidate whether *Chlamydiaceae* MurA resistance may be connected to an ancestral capacity for fosfomycin production. However, it remains to be confirmed if this BGC is indeed used to produce fosfomycin or instead a different phosphonate antimicrobial.

Overall, our results show that many *Chlamydiae* members have the potential to produce secondary metabolites, which may play a previously unrecognized role in their endosymbiotic lifestyles. As far as we are aware, secondary metabolite biosynthesis had not been previously noted or investigated in the *Chlamydiae* phylum. This could be explained by the absence of these gene clusters in the most-studied family, the *Chlamydiaceae*. The *Chlamydiae* phylum is part of the PVC superphylum and other PVC members are also associated with sponges [18, 34]. In particular, *Planctomycetes* phylum members have been identified as a potential reservoir of novel secondary metabolites [68, 69]. Despite *Planctomycetes*-affiliated genomes being substantially larger [69], a comparable number of BGCs were identified in *Chlamydiae*-affiliated genomes (Fig. 5a). The *Chlamydiae* phylum may thus likewise represent a reservoir for the discovery of secondary metabolites.

Based on their endosymbiotic lifestyles and smaller genome sizes, it was unexpected to identify BGCs as common in many *Chlamydiae* genomes. These chlamydial BGCs could function in inter-microbial warfare, in communication, or in mediating host interactions [70]. In addition, chlamydial BGCs could function in providing chemical defence to the host. Members of the sponge microbiome have been suggested to provide chemical defence to the sponge host [5]. Recently, an endosymbiosis was identified between a *Haliclona* sponge species and a gammaproteobacterium mediated by chemical defence through antibiotic production [71]. Sponge-associated chlamydiae genomes that encode BGCs (i.e., *Ca*. Sororchlamydiaceae and MCF-D family members; Fig. 5a) may likewise participate in host-beneficial defensive endosymbioses. MAGs from other putatively endosymbiotic bacteria (e.g., affiliated with *Legionellales* and *Rickettsiales*) were also obtained from the metagenomes (Data S4), and specifically from samples that had high *Chlamydiae* relative abundances (Fig. 1a). This suggests the potential for intracellular interactions between co-infecting endosymbionts, perhaps mediated by secondary metabolites. Alternatively, the co-occurrence of putatively endosymbiotic bacteria in some sponge metagenomes could simply indicate increased susceptibility to infection under certain environmental conditions or in some sponge individuals. Furthermore, it could also suggest co-infection of a different eukaryotic host, whose association with the sponges is in turn variable. Novel antimicrobial compounds have also been isolated from sponges that are active against chlamydial species [72], and could instead indicate antagonistic interactions with the sponge host or members of the sponge microbiome. Some protist-infecting *Parachlamydiaceae* members have previously been shown to be mutualists that protect their host amoeba against *Legionella* infection through an unknown mechanism [73, 74]. This mechanism may have a basis in the production of secondary metabolites as many BGCs were identified in *Parachlamydiaceae*-affiliated genomes (Fig. 5a). Some sponge-associated chlamydiae could offer similar defensive benefits against co-infection to their host.

### *Chlamydiae* is associated with other sponge species and marine invertebrates

**Fig. 6:**
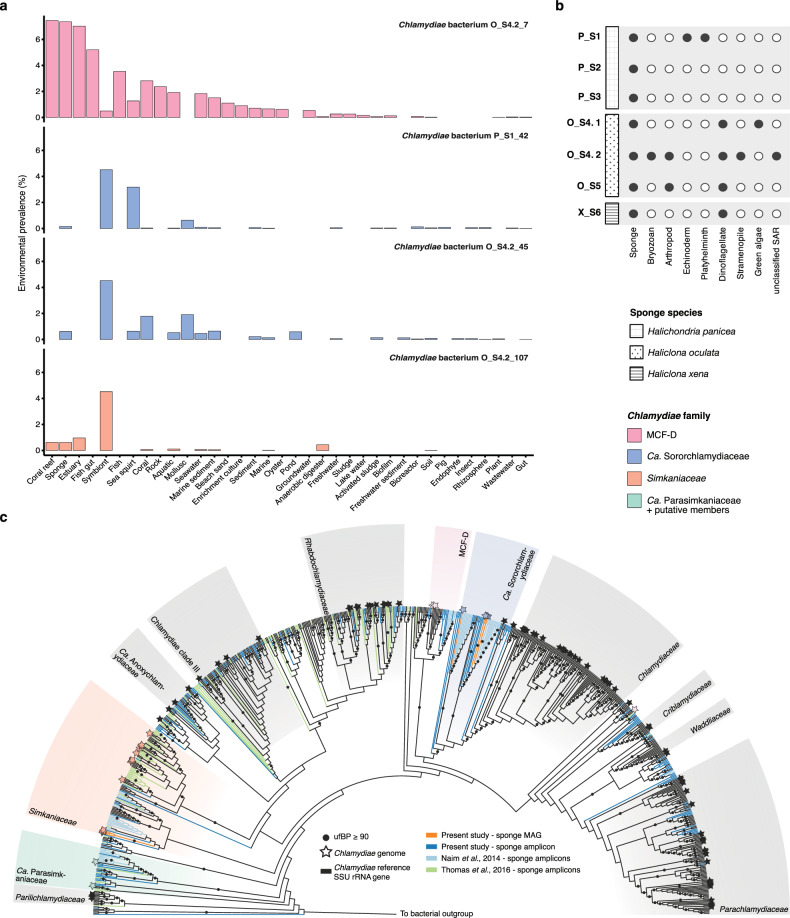
Relatives of sponge-associated chlamydiae are primarily found in marine habitats, and no other putative eukaryotic hosts were identified in the sponge metagenomes based on SSU rRNA genes. **a** Percentage of amplicon samples from various environments with SSU rRNA genes ≥95% identity to the indicated chlamydial sponge MAG. A representative from each chlamydial species group with MAGs that include SSU rRNA genes is shown (Fig. 2a). Only environments with ≥100 samples, and clear labels are shown. See Data S11 for full IMNGS results of SSU rRNA gene searches against NCBI SRA amplicon datasets. **b** Presence of eukaryotic SSU rRNA genes, and their corresponding taxonomy, across sponge sample metagenome assemblies. See Data S4 for full taxonomy, contig IDs, and contig coverage. **c**. Sponge-associated chlamydiae are restricted to specific chlamydial groups. Maximum-likelihood phylogeny of small subunit rRNA genes from across *Chlamydiae* (and outgroup sequences) inferred using the GTR + F + R10 model and shown as a cladogram for clarity (See Data S5 for sequence accessions and branch lengths). Included are reference chlamydial sequences (black), sponge-associated chlamydiae genomes and amplicons from the present study (orange and blue), chlamydial amplicons previously obtained from these sponge species (light blue; Naim et al., 2014) [20], and chlamydial amplicons obtained from a prior study of sponge microbial diversity (green; Thomas et al.) [18]. Sequences from *Chlamydiae* genomes, and thus representing sequenced genomic diversity, are shown by stars. Chlamydial families are coloured according to the legend and labelled.

To determine whether sponge-associated chlamydiae are associated with other hosts or environments we screened publicly available SSU rRNA gene amplicon datasets for close relatives (≥95% identity) (Fig. 6a and Data S11). Relatives of sponge-associated chlamydiae were found almost exclusively in marine environments, with higher prevalence in marine invertebrates such as sponges, corals, sea squirts, and molluscs (Fig. 6a). Sponge-associated chlamydiae affiliated with MCF-D were present in a higher proportion and wider range of environments, yet still primarily of marine origin. Unfortunately, no SSU rRNA gene was obtained for any of the sponge-associated *Ca*. Parasimkaniaceae MAGs, and their environmental distribution could not be assessed. Despite the clear association with marine environments here, *Chlamydiae* have also been found associated with sponges from freshwater lakes [75]. Relatively few environments were identified with higher abundances of sponge-associated chlamydiae relatives (Data S11), possibly due to *Chlamydiae* being undetected and underestimated by many common primers for surveying microbial diversity [25–27]. Those identified included incubations of Great Barrier Reef lagoon water [76] and humic acid amended aquaculture systems (Data S11). In addition to our study, and the previous study of these sponge species [20], *Chlamydiae* have also previously been found in high relative abundance in association with sponges. This includes *Suberites zeteki*, a marine sponge invasive in Hawaii [19], and *H. panicea* after ex situ cultivation [77]. In addition, several chlamydial MAGs have previously been obtained from sponge metagenomes. This includes *Simkaniaceae* bacterium CLI4_bin_1 and *Chlamydiia* bacterium bin105s from the sponges *Cliona orientalis* and *Vazella pourtalesii*, and affiliated with *Simkaniaceae* and MCF-D families (Fig. 2a), respectively [16, 37]. These studies and ours suggest that more chlamydial genomes will likely be uncovered from further metagenomic investigation of sponge-associated microbial communities.

Although *Chlamydiae* has been found across different sponge species and relatives of sponge-associated chlamydiae were detected in various marine invertebrates, it is possible that they instead have another sponge-associated eukaryotic host. To help elucidate this, eukaryotic SSU rRNA genes across the metagenomes were collected and classified (Fig. 6b and Data S4). Some of the identified eukaryotes are present in multiple samples. However, importantly, no other eukaryotes apart from the sponge were found across the samples with high *Chlamydiae* relative abundances (Figs. 1a and 6b). Still, additional eukaryotes may have been missed in the metagenomes, for example due to DNA extraction biases. We did identify a mitochondrial cytochrome c oxidase subunit 1 (CO1) gene most closely related to the green algae *Picochlorum* in samples with high *Chlamydiae* relative abundances, and this could represent an alternative host (Data S4). *Chlamydiae* members have not previously been identified in green algae, which thus far have relatively few known endosymbionts [78]. However, this could be the result of sampling bias. Recently, chlamydiae affiliated with *Simkaniaceae* were detected in some cultures of *Symbiodiniaceae*, a group of dinoflagellate microalgae that are endosymbionts of cnidarians, such as corals [79]. There is thus some precedent for an association between *Chlamydiae* members and algae. In addition, diverse eukaryotes have been found associated with sponges that could act as alternative hosts. For example, eukaryotes found associated with *H. panicea* include diatoms, algae, ciliates, nematodes, turbellaria, amphipods, and copepod and polychaete larvae [80, 81]. *Chlamydiae* has also previously been detected in a sponge-associated polychaete microbiome [82]. Indeed, several *Simkaniaceae*-affiliated MAGs have been retrieved from marine worm metagenomes (Data S6). Fungi were also found in other *H. panicea* and *H. xena* samples from the Oosterschelde estuary, although at low relative abundance [83]. While we found a clear association of some *Chlamydiae* lineages and sponge species, further work is needed to determine whether this is through an alternative eukaryotic host.

*Chlamydiae* was found across a wide range of sponge microbiomes in a recent amplicon survey [18]. This prompted us to examine whether specific chlamydial groups are associated with sponges. To answer this question we inferred a SSU rRNA gene phylogeny using chlamydial sequences obtained from this wide survey [18], from our study, and from the prior study of these sponge species [20]. We then placed them in the context of a representative dataset of bacterial and chlamydial SSU rRNA gene sequences [25, 26], and sequenced *Chlamydiae* species representatives (Data S6). In the resulting phylogenetic tree a separation is apparent, with the vast majority of sponge-associated sequences affiliating with less studied chlamydial families (Fig. 6c). No sponge sequences grouped together with the well-studied *Chlamydiaceae* family, and few sequences affiliated with the protist-infecting *Ca*. Amoebachlamydiales families (i.e., *Criblamydiaceae*, *Waddliaceae*, and *Parachlamydiaceae*) (Fig. 6c). As expected, sequences from the present study and previous study of these sponge species [20] clustered together in the *Ca*. Sororchlamydiaceae, MCF-D, *Simkaniaceae*, and *Ca*. Parasimkaniaceae clades (Fig. 6c). Chlamydial sequences from the wide survey of sponge microbiomes [18] primarily grouped together with the *Ca*. Parasimkaniaceae, *Simkaniaceae*, *Rhabdochlamydiaceae*, and in unclassified clades (Fig. 6c). Altogether, these observations provide evidence for widespread associations between less-studied chlamydial groups and sponges.

### Conclusions and future perspectives

Using genome-resolved metagenomics we have expanded sequenced *Chlamydiae* diversity with 18 high-quality genomes that provide insight into chlamydial associations with animal hosts. This allowed us to uncover genomic diversity from four distinct chlamydial families and to describe two that previously had poor representation, *Ca*. Sororchlamydiaceae and *Ca*. Parasimkaniaceae. All cultivated *Chlamydiae* members are obligate endosymbionts [23] and based on their gene content sponge-associated chlamydiae likely are as well. Sponge-associated chlamydiae are capable of acquiring carbon and energy directly from eukaryotic hosts (e.g., through the action of NTTs and UhpC etc.). Thus, their capacity for degrading a wide range of sponge-derived compounds and compounds present in the larger microbial community was unexpected. The presence of genes for degrading toxins and pollutants, and BGCs in some sponge-associated chlamydiae could suggest host-beneficial effects. More generally, our findings open the door for further exploration of BGCs across *Chlamydiae*, and suggest larger roles for secondary metabolites in endosymbiotic interactions. Relatives of sponge-associated chlamydiae are prevalent in other sponges and marine invertebrates, where they have unknown effects.

We found that sponge-associated chlamydiae vary in their relative abundance across specimens of the same sponge species. This points to important unanswered questions about the nature of sponge-chlamydiae associations. Foremost, it is currently unclear whether the presence of sponge-associated chlamydiae is beneficial or detrimental to the sponge, and if their presence is an indicator of environmental conditions and host health? Future studies are also needed to confirm whether these chlamydiae are directly associated with the sponge host and are endosymbionts, or if their interaction is secondary as symbionts of a different eukaryotic host living in the sponges. This important distinction can be elucidated, for example, through direct experimental evidence from fluorescence in situ hybridization, transmission electron microscopy, or single-cell genomics of sponge cells. Our genomic investigation has brought new insights to sponge-associated chlamydiae and suggests chlamydial effects on sponges, whether direct or indirect, that warrant further investigation. Altogether, our work represents a first step in untangling the potentially wide impacts of *Chlamydiae* on marine ecosystems.

### Description of *Candidatus* Sororchlamydiaceae fam. nov

(So.ror.chla.my.di.a.ce’ae. L. fem. n. *soror* sister; *Chlamydiaceae* taxonomic name of a bacterial family; L. suff. *-aceae* ending to denote a family; *Ca*. Sororchlamydiaceae referring to the close relationship to the bacterial family *Chlamydiaceae*)

The family *Candidatus* Sororchlamydiaceae (formerly *Chlamydiae* Clade IV) represents a distinct monophyletic lineage, sister to *Chlamydiaceae*, supported by concatenated marker gene phylogenies in the present study (Fig. 2a and Fig. S3) and in prior work [27, 31]. *Ca*. Sororchlamydiaceae members share conserved gene content and metabolism, and have larger genome sizes and greater metabolic potential than *Chlamydiaceae* members (Fig. 2b, 3 and Fig. S4–S5) [27].

### Description of *Candidatus* Parasimkaniaceae fam. nov

(Par.a.sim.ka.ni. a.ce’ae. Gr. prep. *para* alike, alongside of; N.L. fem. n. *Simkania* taxonomic name of a bacterial genus; L. suff. *-aceae* ending to denote a family; *Ca*. Parasimkaniaceae referring to the close relationship to the bacterial family *Simkaniaceae*).

The family *Candidatus* Parasimkaniaceae represents a distinct monophyletic lineage supported by concatenated marker gene phylogenies and is closely related to the *Simkaniaceae* family (Fig. 2a and Fig. S3). *Ca*. Parasimkaniaceae members share conserved gene content, and have on average smaller genome sizes and reduced metabolic potential relative to *Simkaniaceae* members (Figs. 2b, 3 and Figs. S4–S5).

## Materials and methods

### Sponge collection and DNA extraction

Six sponge specimens from three sponge species (*Halichondria panicea –* P_S1, P_S2, and P_S3; *Haliclona oculata –* O_S4 and O_S5; *Haliclona xena* – X_S6) were collected from the Oosterschelde estuary in the Netherlands, washed in autoclaved seawater, and stored at −80 °C (Fig. S1 and Data S1). P_S1, P_S2, and O_S4 were sampled at the same timepoint and location as those investigated in a previous study (Fig. S1) [20]. The sponge SSU rRNA gene from each metagenome was used to confirm sponge species identification (Data S1).

For generating SSU rRNA gene amplicons, DNA was extracted from 0.2 g of each specimen using the DNAeasy PowerLyzer Powersoil Kit (QIAGEN) according to manufacturer’s protocols, with DNA elution in water and bead beating with the PowerLyzer 24 homogenizer (MO BIO) at 4000 rpm for 45 s. For O_S4 (O_S4.2), O_S5, and X_S5 this DNA was also used for metagenomic sequencing (Fig. S1). Due to high levels of fragmentation an additional extraction optimized for high-molecular-weight DNA was performed for metagenomic sequencing of P_S1, P_S2, P_S3, and a second O_S4 extraction (O_S4.1) (Fig. S1). Thus, two different DNA extraction methods were performed on sponge specimen O_S4 and both were later sequenced (O_S4.1 and O_S4.2). In the high-molecular-weight DNA extraction, bead beating with the DNAeasy PowerLyser Powersoil Kit (QIAGEN) was used as described above, but with the addition of 0.2 M ethylenediamine tetraacetic acid (EDTA) (1:1 ratio) prior to the lysis step to inhibit DNases. After bead beating, 10% cetyltrimethylammonium bromide buffer (CTAB) (1:4 ratio), 0.5 M NaCl (1:2 ratio), 0.1 M EDTA (1:4 ratio), 10 μL β-mercaptoethanol (100%), and 5 μL proteinase K (600 mAU/mL) were added and the samples were incubated overnight at 56 °C. RNase A was then added (final concentration of 0.3 ng/μL) and the sample incubated for 30 min at 37 °C. Two rounds of chloroform/isoamyl alcohol 24:1 (1:1 ratio) addition, incubation for 2 min at room temperature, centrifugation (10000 × g for 10 min), and transfer of the aqueous phase were then performed. DNA was precipitated using isopropanol (6 h) and pelleted with centrifugation for 15 min at 10000 × g at 4 °C, before being washed twice with 80% ethanol and eluted in water.

### Generation of bacterial SSU rRNA gene amplicons

A two-step PCR approach was used to obtain SSU rRNA gene fragments for amplicon sequencing (Fig. S1), using the bacterial-specific primers S-D-0564-a-S-15 (AYTGGGYDTAAAGNG) and S-D-Bact-1061-a-A-17 (CRRCACGAGCTGACGAC) [84] that capture most chlamydial lineages [27]. HotStarTaq DNA Polymerase (QIAGEN) was used with the following reaction conditions: initial heat activation at 95 °C (15 min), followed by 28 cycles of denaturation at 94 °C (60 s), a step-down to 70 °C (1 s), a ramping rate of 0.4 °C/s to 50 °C for annealing (60 s), and a ramping rate of 0.8 °C/s to 72 °C for extension (60 s), with a final extension at 72 °C (10 min). A second PCR reaction was performed according to the manufacturers’ protocol to obtain sequence libraries with adaptor sequences from the TruSeq DNA LT Sample Prep Kit (Illumina). PCR products were purified using magnetic Agencourt AMPure XP beads (Beckman Coulter) and sequencing performed on the MiSeq System (Illumina) using the v3 chemistry (2 × 300 bp).

Sequence reads were demultiplexed and quality control performed using Cutadapt v. 1.10 [85] to remove remaining adaptor and primer sequences, trim 3’ read ends to a minimum Phred quality score of 10, and remove reads shorter than 100 bp in length. VSEARCH v. 1.11.1 [86] was then used to merge forward and reverse reads (–fastq-minovlen 16), to de-replicate reads (–derep_fulllength), and to obtain centroid OTU clusters at 97% identity. Chimeric sequences were detected and removed using UCHIME [87] with the SILVA123.1_SSUref_tax:99 database [88]. OTUs were taxonomically classified using CREST v. 4.2.1 [89] with the default ‘silvamod138’ database, which is a modified version of the SILVA nr SSU Ref v138 database [88] (Data S2). OTU read counts and relative abundances across samples are available in Data S3.

### Metagenome sequencing and assembly

The Nextera DNA Library Prep Kit (Illumina) was used to prepare sequence libraries with 25 ng of input DNA, followed by sequencing with the NovaSeq6000 System (Illumina) for P_S1, P_S2, P_S3, and O_S4 (O_S4.1), and with the HiSeq2500 System (Illumina) for O_S4 (O_S4.2), O_S5, and X_S6. Quality control of resulting sequence reads was performed to remove adaptors and low-quality sequences using Trimmomatic v. 0.35 [90] with the options: ILLUMINACLIP:TruSeq3-PE.fa:2:30:10 LEADING:3 TRAILING:3 SLIDINGDOWN:4:15 MINLEN:50. Read quality was assessed using FastQC v0.11.4 [91]. Resulting paired sequence reads were then assembled using MEGAHIT v3.13 [92] (--meta --only-assembler), and assembly statistics obtained with QUAST v5.0.2 [93] (Data S1). Protein sequences were predicted using Prodigal v2.6.3 [94]. Barrnap v. 0.9 [95] was used to identify metagenomic SSU rRNA genes, which were classified using the LCAClassifier from CREST v. 3.1.0 [89] using the default modified 'silvamod138' version of the SILVA nr SSU Ref v138 database [88] (Data S4).

### Binning of metagenome-assembled genomes

Differential read coverage was obtained by mapping each set of sequence reads against each assembled metagenome using Bowtie2 v2.2.6 [96]. MAGs from each metagenome were obtained using differential coverage binning with metaBAT 2.12.1 [97], CONCOCT v. 1.1.0 [98], and MaxBin v. 2.2.7 [99] (Fig. S1). The metaWRAP [100] v. 1.2.4 “bin_refinement” module was then used to consolidate resulting bins into hybridized bin sets. The highest quality hybridized or original bin was selected with a cut-off of 70% completeness and 10% redundancy (Data S4). Several assemblies had smaller sizes (P1_S2 and P1_S3) and few MAGs above quality thresholds were obtained for these (Data S1 and S4). Further manual refinement of chlamydial MAGs was performed using anvi’o [101, 102] v.6.2, followed by reassembly with the metaWRAP [100] v. 1.2.4 “reassemble_bins” module, and additional manual curation (Fig. S1).

### Genome characteristics and annotation

*Chlamydiae* MAGs were annotated using Prokka [103] v1.14.6. In addition, protein-coding genes were annotated with the NCBI NR protein database [104], top Blastp hits [105], and Pfam [106] and TIGRFAM [107] database domains identified by InterProScan [108] 5.47-82.0. Comparative genomic analyses were performed between sponge-associated chlamydiae MAGs and a set of *Chlamydiae* species representatives with high-quality genomes. Genome characteristics and genome quality were determined with miComplete [109] v. 1.1.1 using a marker gene-set conserved in complete chlamydial genomes [27] (Data S6). Genes were also assigned to eggNOG [110] 4.5 NOGs at the universal-level using eggNOG-mapper 1.0.3 (“-d NOG”) [111], and KEGG KOs [112] identified using GhostKOALA [113] (*Chlamydiae* representatives) and BlastKOALA (chlamydial genomes from the present study). Genes of interest were identified (Data S8) and COGs were also assigned to COG pathways (Data S9) [114]. AntiSMASH 6 beta [64] was used to identify BGCs, and top hits to MIBiG clusters [115] where found (Data S10). Chlamydial species groups with ≥95% ANI were identified using dRep [116] v3.2.2 “compare” and the options “--SkipMash” and “-sa 0.95”, and only ANI comparisons with 50% query and reference coverage further considered (Data S6).

### Ribosomal protein phylogeny of metagenomic contigs

Microbial community composition was assessed by identifying metagenomic contigs encoding ribosomal proteins (at least 5 of 15), typically found together in a conserved gene cluster [117], using a previously described pipeline [118] (Data S4). Ribosomal proteins from each contig were concatenated and a ML phylogeny inferred using RAxML [119] 8.2.4 with the PROTCATLG model of evolution, and 100 rapid bootstrap replicates (Data S5). Read coverages of these metagenomic contigs were compared to measure relative abundances (Data S4).

### *Chlamydiae* species phylogeny

Protein sequences from 74 single-copy marker genes (Data S7), found conserved across *Chlamydiae* MAGs and PVC species representatives (Data S6), were each aligned using MAFFT-L-INS-i [120] v7.471, and trimmed with BMGE [121] (entropy of 0.6). IQ-TREE [122] v. 1.6.12 was used to infer single-gene trees with 1000 ultrafast bootstraps inferred [123], and using ModelFinder [124] for model selection from LG [125] and LG profile mixture models (C10 to C60) [126], with gamma or free-distributed rates (+G or +R) [127], and with or without empirically determined amino acid frequencies (+F). Trees were manually inspected, divergent sequences removed, and the process repeated where necessary (Data S7). To determine which marker genes had the strongest phylogenetic signal, the monophyly of PVC phyla in the single-gene trees was assessed (Data S7). Four marker gene datasets were chosen to include all genes (74 NOGs), genes where *Chlamydiae* was monophyletic (60 NOGs), genes where *Chlamydiae* and most other PVC phyla were monophyletic (40 NOGs), and genes where all PVC phyla were monophyletic (15 NOGs) (Data S7). ML species phylogenies were then inferred as described above for concatenated alignments of each dataset.

The topology across all species trees was consistent and the placement of sponge-associated chlamydiae MAGs supported (Fig. S3 and Data S5). Further analyses were thus performed with only the smallest concatenated dataset (15 NOGs). A ML tree was inferred using IQ-TREE [122] v. 1.6.12 with 100 non-parametric bootstraps under the PMSF approximation [128] of the LG + C60 + F + R4 model of evolution (Fig. 2a and Data S5). A Bayesian phylogeny was also inferred using PhyloBayes-MPI [129] 1.7b and the CAT + GTR + Γ4 model of evolution [130], with four independent MCMC chains. After approximately 100,000 generations the four chains had not converged (with a burn-in of 5000, and sampling every 10 generations) (Data S5). However, two chains had converged (maxdiff of 0.16) (Fig. 2a), and the topology of both deeper nodes and the placement of sponge-associated chlamydiae was consistent across all chains (Data S5).

### Single-protein phylogenies of specific genes

The eukaryotic affiliation of typically eukaryotic genes was confirmed using a phylogenetic workflow (https://github.com/jennahd/HGT_trees) (Data S5). Additionally, phylogenetic trees were inferred for protein sequences with SnoaL-like PKS (PF07366) and PepM (PF13714) protein domains (Data S5). Here, DIAMOND Blastp [131] v0.9.36 searches (with “max-target-seqs 2000” and “more-sensitive”) were performed against NCBI’s NR database [104], and sequence redundancy removed using CD-HIT [132] v. 4.8.1 at 80% identity. For PepM, the top 100 hits to the Swiss-Prot database, of proteins with curated annotations, were additionally retrieved [133]. Sequences were aligned and trimmed as above. For SnoaL-like PKS an initial tree was inferred using FastTree2 [134] v2.1.11 and a subset of more distantly related sequences removed. ML phylogenies were then inferred using IQ-TREE [122] v.1.6.12, with model selection by ModelFinder [124] as described above. The LG + C60 + F + R4 model was selected in both cases, and 1000 ultrafast bootstraps inferred [123] (Data S5).

### Small subunit rRNA gene phylogeny of sponge-associated *Chlamydiae* diversity

Chlamydial SSU rRNA gene amplicon OTUs were collected from the present study, the prior study of these sponge species [20], and from a survey of sponge microbial diversity [18]. These were combined with full-length and near full-length SSU rRNA genes from *Chlamydiae* MAGs and reference genomes (Data S6), and prior surveys of chlamydial diversity [25]. Sequences were added to a previously published bacterial SSU rRNA gene alignment of 85% sequence identity representatives [26] using MAFFT-L-INS-i [120] v7.471 (“--add” for near full-length sequences, and “--addfragments” for amplicon OTUs). The alignment was trimmed using trimAl [135] v1.4.rev15 with a gap threshold of 0.1. A ML phylogeny was then inferred using IQ-TREE [122] v. 1.6.12, with the GTR + F + R10 model selected from GTR models [136] by ModelFinder [124] and 1000 ultrafast bootstraps [123].

### Environmental distribution

The environmental distribution of specific chlamydial lineages was assessed using IMNGS [137]. Here, environmental samples were screened for sequences with at least 95% identity to SSU rRNA genes from several sponge-associated chlamydiae MAGs (Data S11). Samples with at least 0.1% relative abundance of these chlamydial lineages were also identified (Data S11).

### Data visualization and availability

Phylogenetic trees were visualized using the ETE3 Toolkit [138], iTOL [139], and Figtree v1.4.4 (http://tree.bio.ed.ac.uk/software/figtree/). Plots were generated using R version 4.0.3 (R Core Team, 2020), with ggplot2 [140] v. 3.3.2, and with UpSetR [141] v. 1.4.0 for intersection plots. Assembled metagenomes, metagenome sequence reads, amplicon sequence reads, and MAGs generated from each sponge sample can be found deposited under BioProject PRJNA742377. Accessions for data analyzed or obtained in this study can be found in Data S1, S4, and S6. Whole Genome Shotgun projects for sponge metagenome assemblies P_S1, P_S2, P_S3, O_S4.1, O_S4.2, O_S5, and X_S6 have been deposited at DDBJ/ENA/GenBank under the accessions JAHZIM000000000, JAHZIN000000000, JAHZIO000000000, JAHZIP000000000, JAHZIS000000000, JAHZIQ000000000, and JAHZIR000000000, respectively. The versions described in this paper are versions JAHZIM010000000, JAHZIN010000000, JAHZIO010000000, JAHZIP010000000, JAHZIS010000000, JAHZIQ010000000, and JAHZIR010000000. All sequence alignments and phylogenetic trees in Newick format, sponge-associated chlamydiae genome annotations, antiSMASH and eggNOG-mapper output files, and KEGG KOs across *Chlamydiae* species representatives are provided at the figshare data repository: 10.6084/m9.figshare.14939475.v1.

## Supplementary information

Supplementary Information

Data S1

Data S2

Data S3

Data S4

Data S5

Data S6

Data S7

Data S8

Data S9

Data S10

Data S11
