# Supplementary material for: Genomic diversity and biosynthetic capabilities of sponge-associated chlamydiae
Source: ISME J. 2022 Aug 30;16(12):2725–40. doi: 10.1038/s41396-022-01305-9 (PMC9666466; doi:10.1038/s41396-022-01305-9)
Supplement: Supplementary file 1 — Supplementary Information [file 41396_2022_1305_MOESM1_ESM.pdf]

Genomic diversity and biosynthetic capabilities of sponge-associated chlamydiae

Jennah E. Dharamshi[1,*], Natalia Gaarslev[1], Karin Steffen[2], Tom Martin[1], Detmer Sipkema[3], Thijs J. G. Ettema[3,*]

[1] Department of Cell and Molecular Biology, Science for Life Laboratory, Uppsala University, SE-75123 Uppsala, Sweden
[2] Department of Pharmaceutical Biosciences, Biomedical Center, Uppsala University, SE-75123 Uppsala, Sweden
[3] Laboratory of Microbiology, Wageningen University and Research, 6708 WE Wageningen, The Netherlands

*Corresponding authors, thijs.ettema@wur.nl and jennah.dharamshi@gmail.com

SUPPLEMENTARY FIGURES

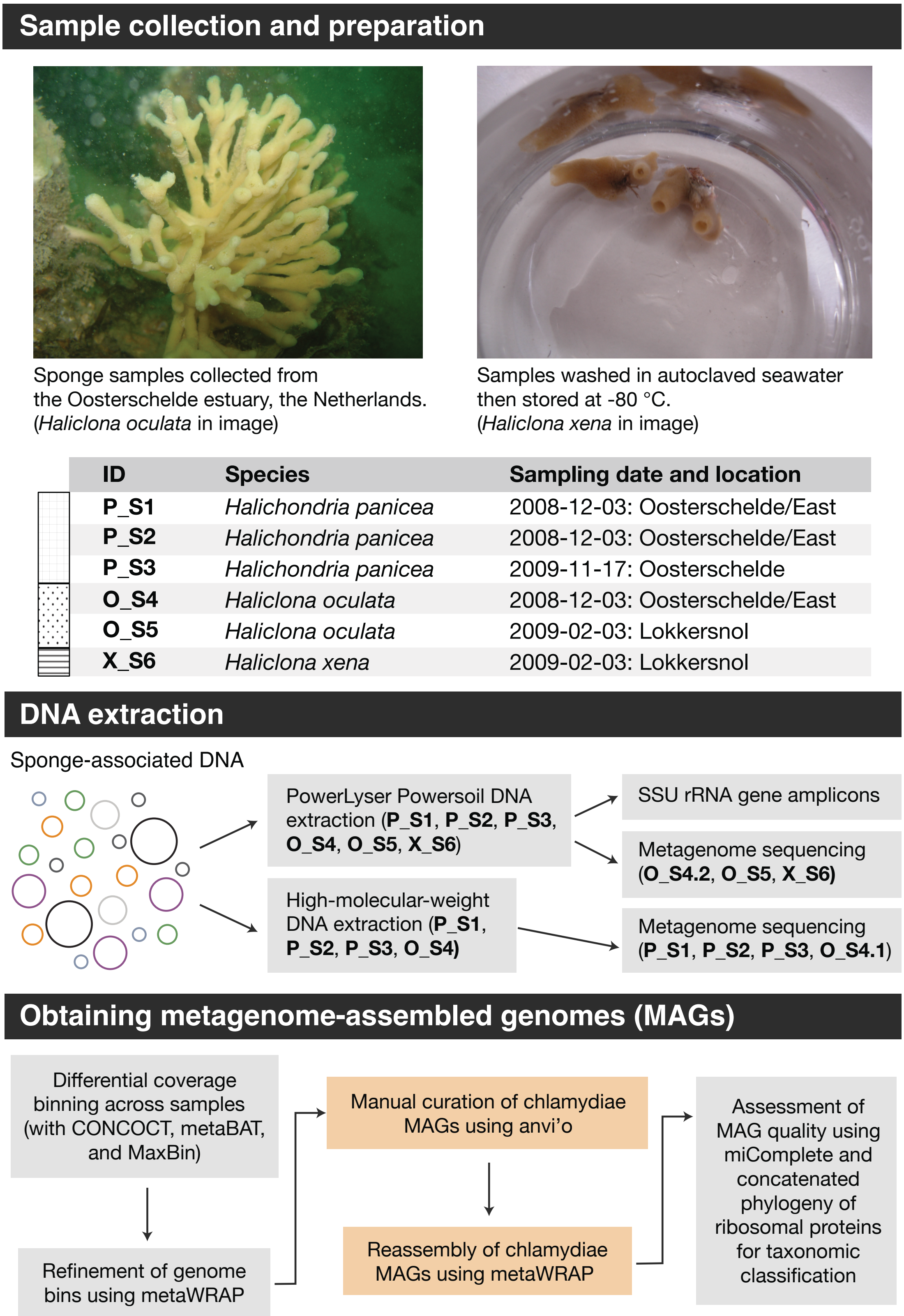

Figure S1. Methods overview of sample collection, sponge specimens, DNA extraction methods, amplicon sequencing, metagenome sequencing and assembly, and metagenome-assembled genome (MAG) binning and manual refinement of sponge-associated chlamydiae MAGs.

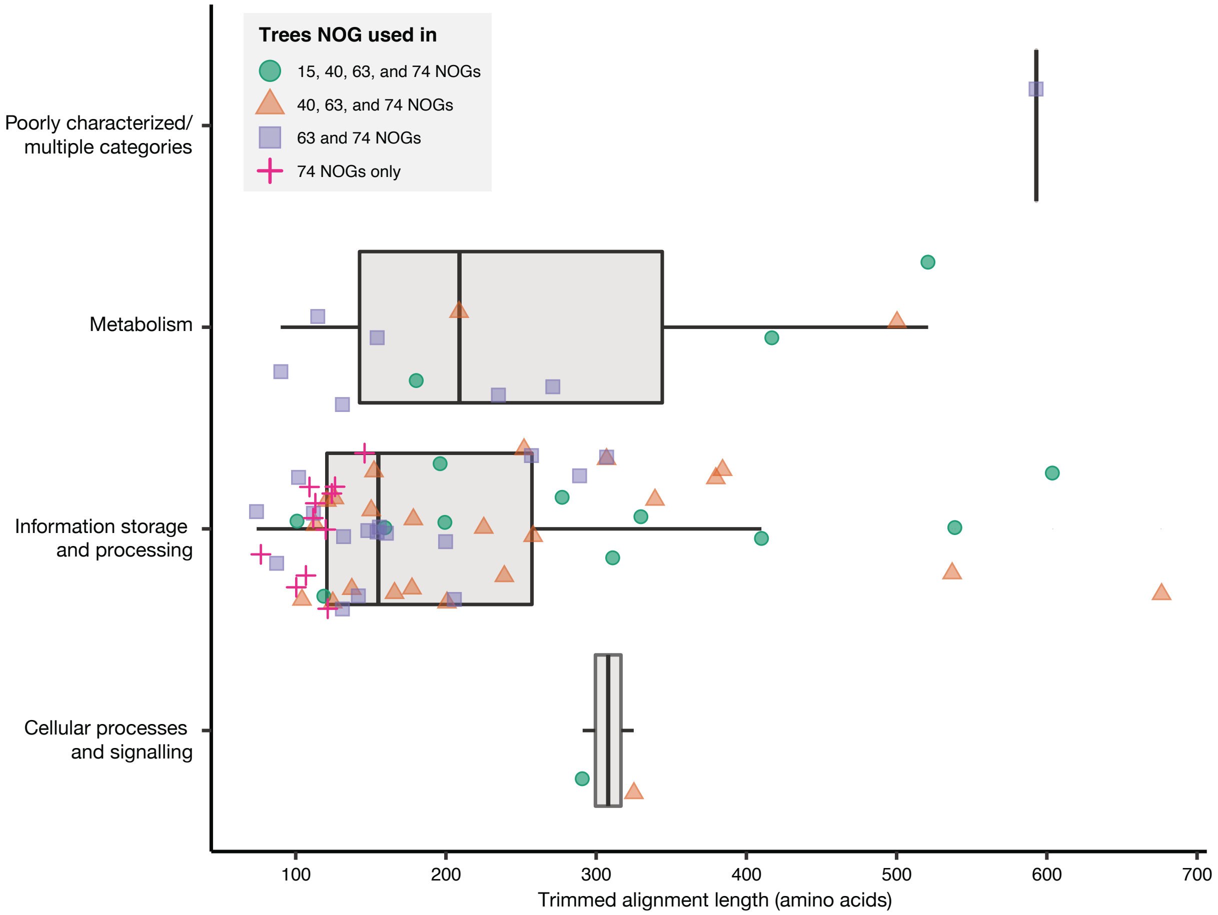

Figure S2. COG category membership of the 74 marker gene NOGs included in *Chlamydiae* species tree concatenated datasets alongside their trimmed alignment lengths. NOGs were assigned to datasets based on phylogenetic signal, measured by resolving *Chlamydiae* and other PVC phyla as monophyletic in single-protein trees. All marker gene NOGs were included in the 74 NOG dataset, those resolving *Chlamydiae* as monophyletic in the 63 NOG dataset, those resolving *Chlamydiae* and most PVC phyla as monophyletic in the 40 NOG dataset, and those resolving all PVC phyla as monophyletic in the 15 NOG dataset. See Data S7 for tree refinement and selection details.

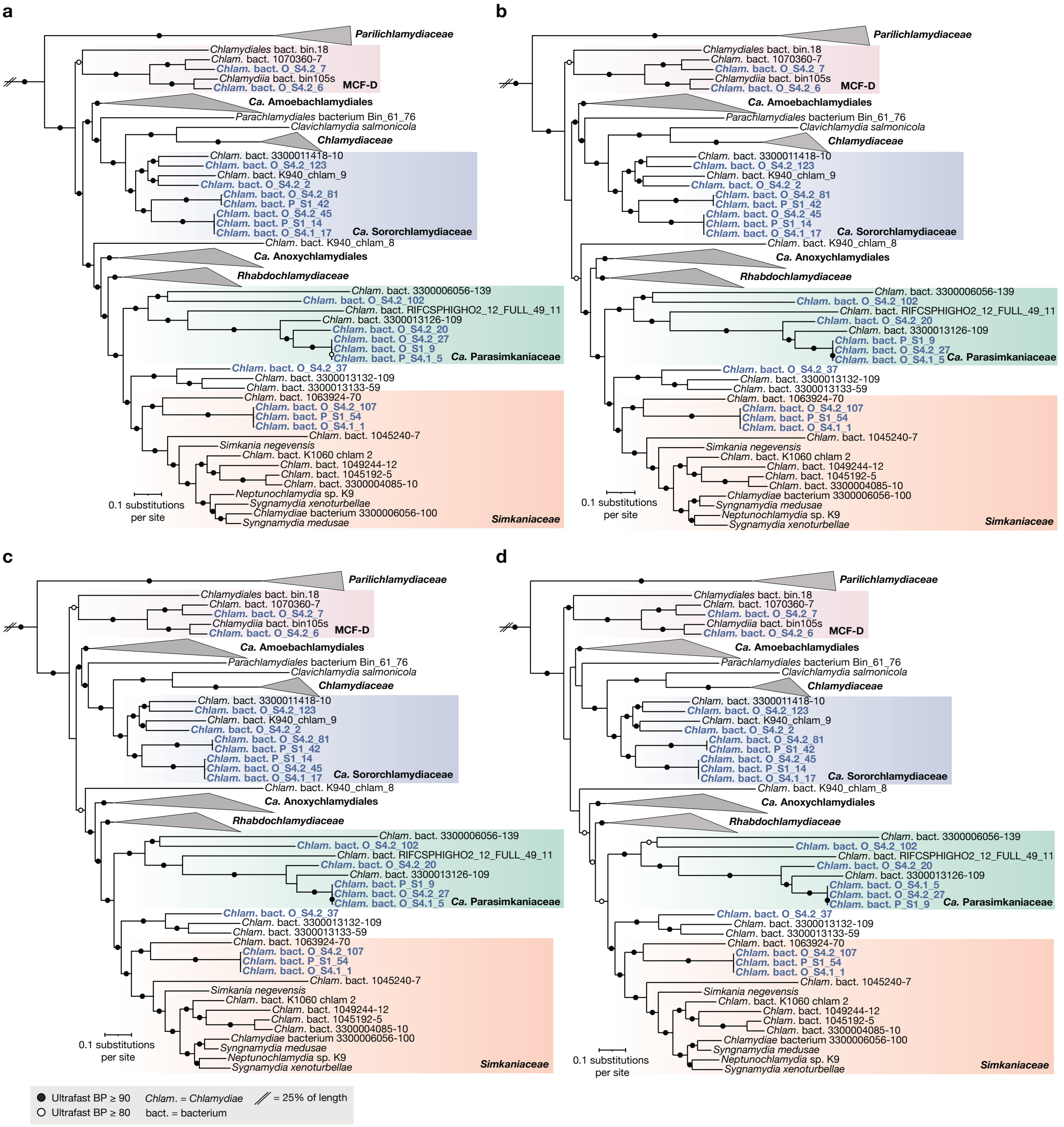

Figure S3. The topology of *Chlamydiae* species relationships is consistent across reconstructions using subsets of the larger initial dataset of 74 NOGs. Concatenated maximum-likelihood protein phylogenies of *Chlamydiae* species inferred under the LG+C60+F+R4 model of evolution with 74 (**a**), 63 (**b**), 40 (**c**), and 15 (**d**) single-copy marker NOGs with 16760, 15502, 11211, and 4757 amino acid alignment positions, respectively. Trees are rooted by a PVC bacteria outgroup (not shown), with this branch reduced to 25% of length. Sponge-associated chlamydiae MAGs are coloured in blue and relevant families coloured. Ultrafast bootstrap (BP) branch support is indicated by circles according to the legend. See Data S5 for uncollapsed species trees and Data S7 for NOGs included in each phylogenomic dataset.

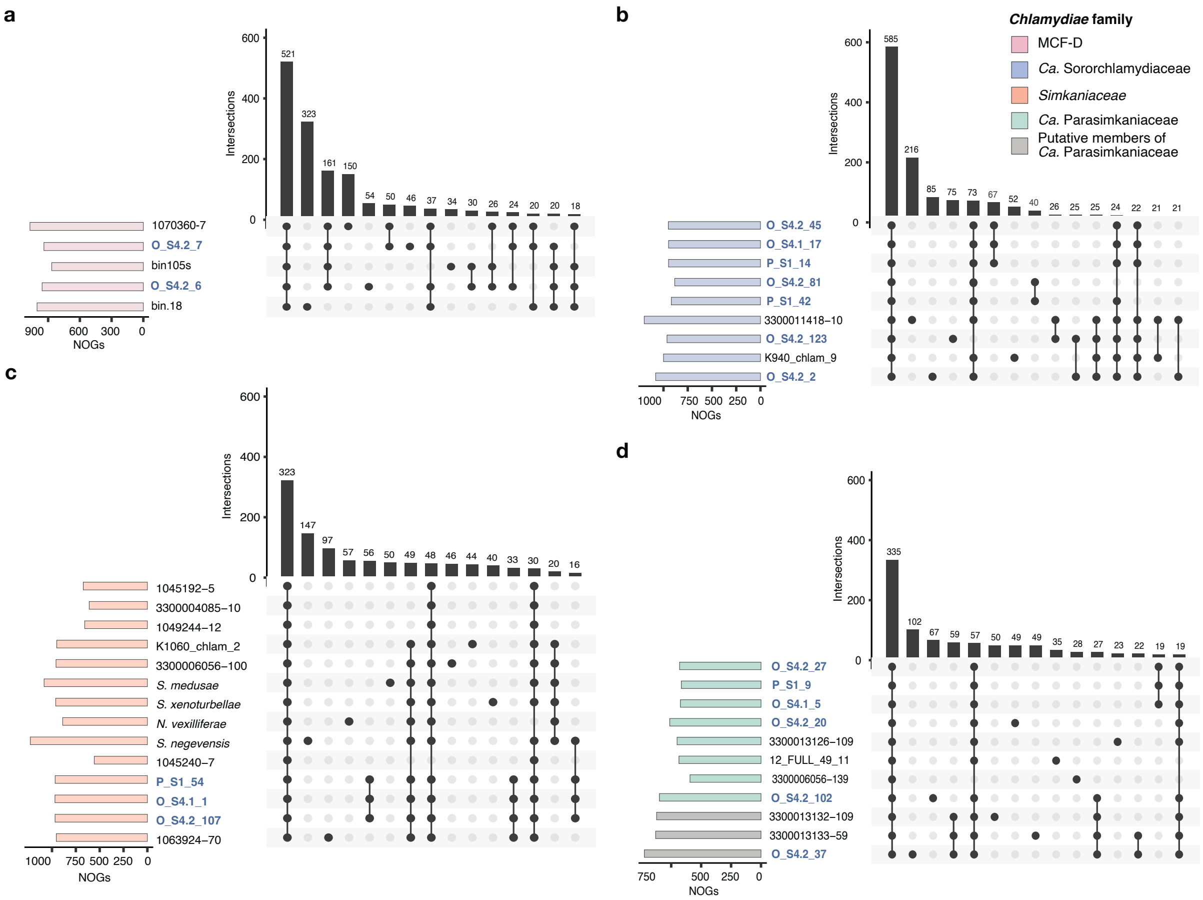

Figure S4. *Chlamydiae* families share varying levels of core gene content, with smaller sets of genes specific to sub-groups of sponge-associated chlamydiae MAGs. Intersection plots give an overview of shared gene content across chlamydiae genomes from the families MCF-D (**a**), *Ca.* Sororchlamydiaceae (**b**), *Simkaniaceae* (**c**), and *Ca.* Parasimkaniaceae (**d**). Intersections are based on sets of NOGs found in each genome, with the total number of identified NOGs found in bar charts to the left of each taxon. Each intersection plot shows the number of NOGs shared between the taxa indicated by black circles for the top 15 most abundant intersections.

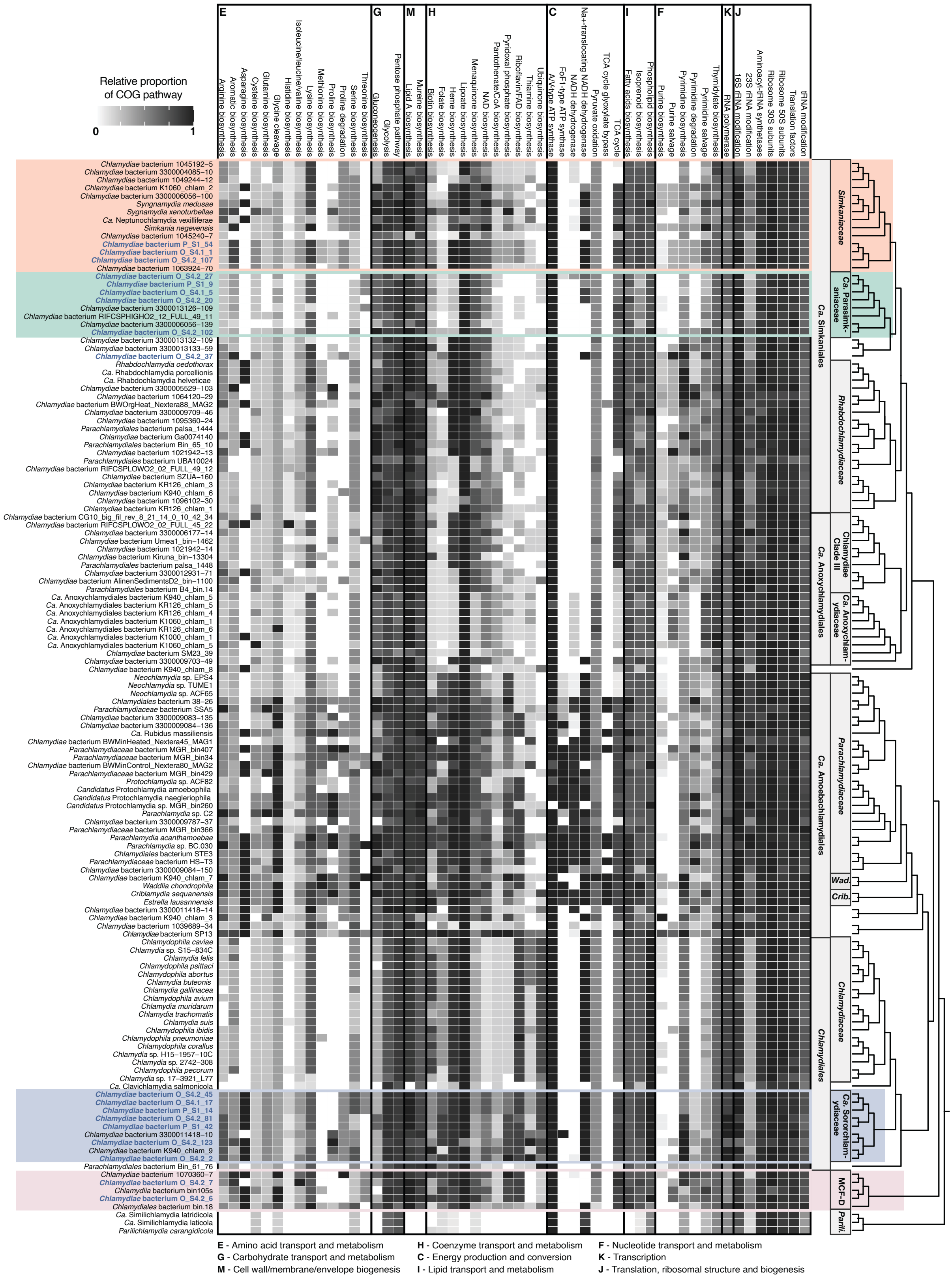

Figure S5. Overview of clusters of orthologous groups (COGs) pathways across *Chlamydiae* indicate that sponge-associated chlamydiae have similar metabolic profiles to other members of their respective families. The heatmap is organized by COG category and shows the proportion of COGs from a pathway found across taxa, relative to the maximum among chlamydiae. Sponge-associated chlamydiae MAGs are in blue, with relevant families coloured accordingly. Order and family names are indicated to the right alongside a cladogram of species relationships, with the following short forms: *Criblamydiaceae* (Crib.), *Parilichlamydiaceae* (Parili.), and *Waddliaceae* (Wad.). See Data S9 for an overview of each COG across Chlamydiae genomes.

SUPPLEMENTARY DATA

Data S1. Sponge sample metadata and NCBI sequence accessions (A), metagenome assembly statistics (B), and confirmation of sponge identity based on metagenomic SSU rRNA genes (C).

Data S2. Bacteria-specific SSU rRNA gene amplicon OTU sequences in FASTA format.

Data S3. Overview of SSU rRNA gene amplicon OTU relative abundances (A), read counts (B), and overall relative abundances of key taxonomic groups (phyla and classes) (C).

Data S4. Sponge metagenome microbial diversity and MAG information. Sequence IDs, coverage, and taxonomy of metagenomic contigs encoding SSU rRNA genes (and corresponding amplicon OTU) (A) and ribosomal proteins (B). Overview of medium to high quality MAGs retrieved from each metagenome, including NCBI sequence accessions, genome characteristics, and taxonomic classification (C).

Data S5. Uncollapsed phylogenetic trees included in this study: ribosomal protein phylogeny of metagenomic contigs, species phylogenies of concatenated marker proteins, single protein phylogenies including phosphoenolpyruvate mutase and SnoaL-like polyketide cyclase, and the sponge-associated chlamydial SSU rRNA gene phylogeny. See page 1 for overview of trees.

Data S6. Genome characteristics, IDs, and NCBI sequence accessions of *Chlamydiae* species representatives and refined sponge-associated chlamydiae genomes (A), and PVC bacteria species representatives (B) used in species phylogenies and comparative genomic analyses. Average nucleotide identity comparisons between chlamydiae genomes (C).

Data S7. Single-copy marker proteins used in concatenated species phylogenies, sequences removed during refinement, and monophyly of PVC phyla in each single-protein tree (A).

Data S8. Overview of sponge-associated chlamydiae enriched and endosymbiotic-lifestyle associated genes across *Chlamydiae* alongside the number of respective sequences in each NOG or KEGG KO gene ortholog (A). Corresponding gene annotations from sponge-associated chlamydiae MAGs examined here including Pfam domains (B).

Data S9. Overview of the presence and absence of COGs from each COG pathway across *Chlamydiae* (A), and annotations of the corresponding genes from sponge-associated chlamydiae MAGs (B).

Data S10. Overview of antiSMASH results with the type and number of BGCs identified in each chlamydiae species representative genome, including sponge-associated chlamydiae MAGs (A). BGCs with top MiBIG hits are indicated alongside top hit information including similarity scores (B).

Data S11. Prevalence of close relatives of sponge-associated chlamydiae (95% identity) across environmental samples based on identification in NCBI SRA SSU rRNA gene amplicon datasets available in IMNGS (A). SRA samples with at least 0.1 % relative abundance (B).
